# Mendelian Susceptibility to Mycobacterial Disease: The First Case of a Diagnosed Adult Patient in the Czech Republic

**DOI:** 10.1155/2020/8836685

**Published:** 2020-12-19

**Authors:** Miroslav Prucha, Hana Grombirikova, Pavel Zdrahal, Marketa Bloomfield, Zuzana Parackova, Tomas Freiberger

**Affiliations:** ^1^Department of Clinical Biochemistry, Hematology and Immunology, Na Homolce Hospital, Prague, Czech Republic; ^2^Centre for Cardiovascular Surgery and Transplantation, Brno, Czech Republic; ^3^Medical Faculty, Masaryk University, Brno, Czech Republic; ^4^Department of Vascular Surgery, Na Homolce Hospital, Prague, Czech Republic; ^5^Department of Immunology, Motol University Hospital and Second Faculty of Medicine, Charles University, Prague, Czech Republic; ^6^Department of Pediatrics, Thomayer's Hospital and First Faculty of Medicine, Charles University, Prague, Czech Republic

## Abstract

We present a case of a 42-year-old woman with Mendelian susceptibility to mycobacterial disease. The disease was diagnosed at an adult age with relatively typical clinical manifestations; the skeleton, joints, and soft tissues were affected by nontuberculous mycobacteria: *Mycobacterium lentiflavum*, *M. kansasii*, and *M. avium*. A previously published loss-of-function and functionally validated variant NM_000416.2:c.819_822delTAAT in *IFNGR1* in a heterozygous state was detected using whole-exome sequencing. After interferon-*γ* therapy was started at a dose of 200 *µ*g/m^2^ three times a week, there was significant clinical improvement, with the need to continue the macrolide-based combination regimen. In the last 4 months, she has been in this therapy without the need for antibiotic treatment.

## 1. Introduction

Mendelian susceptibility to mycobacterial diseases (MSMD) belongs to the group of primary immunodeficiencies, i.e., a: defects in intrinsic and innate immunity. b: MSMD and viral infection: VI-IUIS update [1]. It is a primary immunodeficiency denoted by molecular defects in the interleukin 12 (IL-12)/interferon *γ*- (IFN-*γ*-) dependent signalling pathway. The genetic aetiology of the disease was first demonstrated in 1996 [2], and we currently know of variants in 15 genes that, due to allelic heterogeneity, are the cause of 21 forms of the disease [3, 4]. Patients with this primary immunodeficiency are characterized by a narrow vulnerability to poorly virulent mycobacteria, such as bacillus Calmette–Guérin (BCG) vaccines and environmental mycobacteria (EM) [5, 6]. IFN-*γ* is a key player in driving anti-infectious immunity. It is capable of enhancing antigen processing, inducing an antiviral state, and boosting antimicrobial functions. Severe recurrent infections, either disseminated or localized, are typical here [7].

## 2. Case Report

A 42-year-old female with a suspicion of “immunodeficiency” came to the immunology clinic. The reason at the time was a present active infection by atypical mycobacteria that affected the skeleton with multiple defects of the spine (Th and L vertebrae), skin, and subcutis of the face, lymph nodes, knee joints, calva, paranasal sinuses, and nose. Immunological investigation revealed normal IgG, IgG subclasses, IgM, and IgA levels. Antibody titres to protein (tetanus, diphtheria) and polysaccharide (pneumococcal) vaccine antigens were normal. The patient had normal lymphocyte subset values, and an oxidation test was normal. A summary of laboratory findings on her first visit to our hospital is presented in Table 1.

The patient's history was long, with the onset of clinical problems in childhood. The chronology of the patient's clinical manifestations is included in Table 2. BCG vaccination in the first month of her life did not cause any immediate complications; however, at 5 years of age, tuberculosis of the lymph node developed, and conventional antituberculous therapy was initiated, i.e., isoniazid and rifampicin were administered. The therapy, which lasted 8 months, was successful and was followed by a long period of remission without any clinical manifestations. The patient was free of clinical complaints until 16 years of age, when an interstitial lung disease was diagnosed. After an erroneous diagnosis of sarcoidosis, the patient was treated with systemic glucocorticoids. During this therapy, dysbacteriosis was clinically manifested. It affected the skin, skeleton, and soft tissues. NTM species were identified using microbiological cultivation methods and subsequently treated according to the proven sensitivity. HIV infection was excluded. From that time, the patient was treated continuously with orally and then parenterally administered antituberculosis drugs (rifampicin, isoniazid, pyrazinamide, streptomycin, and ethambutol) for *Mycobacterium lentiflavum* infection that affected the nasal wings and nasal septum. Th8, Th11, L1, L3, and L5 discitis were etiologically caused by *Mycobacterium kansasii*. The knee joints were affected with *Mycobacterium kansasii* and *Mycobacterium avium intracellulare*.

In view of the clinical picture, we suspected a diagnosis of MSMD. Whole-exome sequencing performed on the NextSeq Illumina platform uncovered a frameshift variant NM_000416.2:c.819_822delTAAT; p.Asn274Hisfs *∗* 2 in the heterozygous state in exon 6 of the *IFNGR1* gene coding IFN-*γ* receptor 1, which was confirmed using Sanger sequencing. In addition, a heterozygous sequence variant NM_052813.4; c.1434 + 1G > *C* in the *CARD9* gene was detected. The patient was also submitted to the following laboratory tests: The ability of the proband's CD4 + and CD8 + T cells to produce IFN-*γ* after nonspecific stimulation with phorbol myristate acetate (PMA) was undisturbed. The proband showed markedly increased expression of IFN*γ*R1 on monocytes, myeloid dendritic cells, and plasmacytoid dendritic cells compared with a healthy control. STAT1 phosphorylation (pSTAT1, Tyr701) assay after IFN-*γ* stimulation was performed to assess IFN*γ*R1 downstream signalling. The pSTAT1 response was decreased but not absent, compared with a healthy control. Thereafter, the patient was treated with rhIFN-*γ* and showed significant clinical improvement. rhIFN-*γ* (Imukin, Boehringer Ingelheim) was started at a dose of 200 *μ*g/m^2^ subcutaneously three times per week. The IFN-*γ*-1b therapy was continued, together with antituberculous treatment. The patient's clinical status stabilized. In the last 4 months, she has been in this therapy without the need for antibiotic treatment.

## 3. Discussion

MSMD is a rare disease, despite an increase in its incidence [3, 8], that belongs to the category of primary immune deficiencies. The prevalence of this disease is unknown. The affected immunological mechanism involves the Th1 pathway, namely, mononuclear phagocytes and IFN-*γ* immunity. Localized or systemic infections with nontuberculous mycobacteria are the main clinical manifestations. The defence against mycobacterial infection is mediated by the following mechanism under physiological conditions. Infected mononuclear phagocytes produce IL-12, which stimulates *T* and natural killer (NK) cells to produce IFN-*γ*. Upon binding to receptors, IFN-*γ* stimulates macrophages to produce IL-12, tumour necrosis factor *α* (TNF-*α*), and IL-1. Activated macrophages then kill intracellular pathogens, while activated Th1-phenotype T cells proliferate and release IFN*-γ*. The secreted TNF-*α* then plays a critical role in the formation of granulomas [9]. In the case of an inherited defect of the IFN-*γ* receptor, this defensive pathway is disrupted. One of the main groups of etiological agents found with MSMB patients are nontuberculosis bacteria. Currently, more than 170 NTM species are known in clinical practice with different degrees of pathogenicity and importance [10]. A survey of NTM treatment has recently been published [11].

In addition to atypical mycobacteria, the intracellular agent is employed etiologically, where the functionality of the interferon defensive pathway is essential for immunity. These include *Salmonella* spp., *Listeria* spp., leishmaniasis, *Candida*, histoplasmosis, coccidioidomycosis, HHV8, respiratory syncytial virus (RSV), and vesicular stomatitis virus (VSV). For MSMD diagnostics, clinicians can use the combination of different diagnostic approaches, but most have limitations [12]. Genetic analysis is the most powerful approach to a full diagnosis. Our patient carried a heterozygous variant c.819_822delTAAT in the *IFN-γ receptor1* gene, which was evaluated by the ClinVar database as pathogenic.

Jouanguy et al. [13] considered c.819 a small deletion hotspot with two repeats in close proximity, which could cause slipped strand mispairing, further leading to deletion. This variant was repeatedly reported in patients suffering from mycobacterial infection [14–17], and it was confirmed that the variant completely disrupts expression of the receptor [17]. While immunodeficiency caused by *IFNGR1* variants can be inherited in both autosomal recessive (Immunodeficiency 27A, Phenotype MIM number 209950) and autosomal dominant modes (Immunodeficiency 27B, Phenotype MIM number 615978), all pathogenic variants identified so far in exon 6 have led to mycobacterial infection susceptibility with an autosomal dominant mode of inheritance [18]. These nonsense and frameshift variants lead to premature termination of protein synthesis, but they do not affect the IFN-*γ* binding site and transmembrane domain, so the mutated protein is still capable of binding to the membrane. However, these defects disrupt signalling through STAT1 and internalization of the protein, which results in accumulation of defective IFNGR1 on the cell membrane [19]. Indeed, we observed an increased amount of IFNGR1 expressed on monocytes and dendritic cells, as well as decreased STAT1 signalling, after IFN-*γ* stimulation in our patient. As the IFNGR complex comprises two IFNGR1 chains, the variants exert a double-negative effect, which is further emphasized by increased functional IFNGR1 binding to accumulated defective IFNGR1. Still, a small fraction of the IFN-*γ* receptor is functional, and the disease is manifested as a partial deficiency, typical for Immunodeficiency 27B [19].

In addition, the c.1434 + 1G > *C* variant in the *CARD9* gene has been shown to disrupt mRNA splicing, thus resulting in out-of-frame exon 11 skipping and production of a truncated, but stable, protein [20]. However, this protein cannot be activated by TRIM62. This variant functions in a double-negative manner, reducing cytokine production by dendritic cells and acting protectively against inflammatory bowel disease [21]. Although Szymanski et al. [22] suggested this variant to be associated with an increased risk of pulmonary nontuberculous mycobacterial infection, and the evidence supporting its role in MSMD development is missing. We do not consider this variant causative for MSMD; however, its contribution to the patient's phenotype cannot be excluded.

It is necessary to remark that whole-genome sequencing has its limitations. One of the causes of MSMD may be the presence of anti-IFN gamma autoantibodies, in adults in particular. It is an important differential diagnosis which cannot be addressed by whole-exome sequencing. Laboratory tests are very useful in establishing the concentration of gamma interferon. Its high concentration prompts the suspicion of complete IFNG receptor deficiency [23]. Both functional testing and whole-exome sequencing are, therefore, important.

We were not able to assess our patient's family history. Supposedly, her mother had similar problems but refused examination. The patient has no siblings or children. NTM isolates identified in our patient are typical for MSMD  and in agreement with other published studies [24].

In our patient's history, two interesting moments should be mentioned. (i) She was vaccinated against tuberculosis without complications, but at 5 years of age, she developed dysbacteriosis of the lymph nodes. However, the therapy was successful, and there was a long period of remission without any clinical manifestations. (ii) She took a turn for the worse after the use of glucocorticoids at 17 years of age for suspected sarcoidosis. We believe that the diagnosis was incorrect. It was not sarcoidosis but initial signs of dysbacteriosis, which subsequently manifested clinically upon administration of systemic corticosteroids. Clinical problems with affected bones are typical of AR IFNGR1 deficiency [25]. From the patient's perspective and from a practical point of view, it is important that, by using IFN-*γ* therapy, we significantly influenced the clinical manifestations of the disease and improved our patient's quality of life. Although it was not possible to discontinue the use of antituberculous agents immediately, it was not necessary to continue their parenteral administration. At present, the patient has already been without antibiotic therapy and any clinical problems for 2 months. IFN-*γ* therapy may be useful for some causes of MSMD [26, 27]. However, the response to IFN-*γ* treatment is variable, and the mechanism of action remains unclear. There is a lot of uncertainty as far as  MSMD IFN-*γ* is concerned. Apart from not knowing the exact mechanism of the effect, we are lacking sufficient data about the dose which should be administered. Various schemes are used, the general principle being an increased dose in case of an insufficient effect. We applied a higher dose of IFN-*γ* with our patient as a result of the duration and seriousness of her clinical manifestations. At present, the patient does not exhibit any positive clinical manifestations or positive microbial diagnostics. It should be noted that, for autosomal recessive (AR) complete IFN-*γ* receptor deficiency, this therapy is not effective [28]. Clinically, the characterization of IFNGR1 deficiency-associated variants translates to important differences in the treatment approach. Complete autosomal recessive IFNGR1 deficiency is characterized by early onset of disseminated life-threatening infections by low-virulent mycobacteria, a lack of response to IFN-*γ* cytokine replacement therapy, and high mortality [29]. There appears to be some effect for AD partial IFN-*γ* receptor1 deficiency, IL-12 receptor beta1 deficiency, and IL-12p40 deficiency. The most important points of our casuistry are the following: Diagnosis of the primary immunodeficit must be considered, even in patients with atypical infections, which need not be manifested in the first years of life. Clinically active and inactive periods of disease may take turns. Further extension of the spectrum of these diagnoses with clinically different phenotypes can be supposed. Molecular biology methods, i.e., whole-gene sequencing, represent a fast and reliable way of diagnosing such patients. IFN-*γ* treatment is an example of personalized treatment with some of them, even though the mechanism of its effect is not entirely clear.

Summary: primary immunodeficiency can be manifested clinically at an adult age; this must, therefore, be born in mind during its diagnosis. MSMD is a disease that significantly affects the patient's quality of life. Diagnosis is sometimes difficult due to the low prevalence of this disease. Diagnostics using massive parallel sequencing provides clinicians the opportunity to correctly diagnose these patients and allows targeted therapy that positively affects the development of the disease and the patient's quality of life.

## Figures and Tables

**Table 1:** Summary of laboratory findings.

Summary of laboratory findings	
*Test*	Patient's result
White cell count x10^9^/l (4.0–10.0)	6.2
Neutrophil cells/mm^3^	4100
Lymphocyte cells/mm^3^	1800
IgA g/l (0.7–4.0)	2.03
IgG g/l (6–16)	13.7
IgM g/l (0.5–2.3)	1.25
IgG1 g/l (4.05–10.1)	7.9
IgG2 g/l (1.65–7.85)	4.12
IgG3 g/l (0.11–0.85)	0.52
IgG4 g/l (0.08–1.4)	1.08
CD3 + cells/mm^3^ (700–2100)	1200
CD3 + CD4 + cells/mm^3^ (200–900)	850
CD3 + CD8 + cells/mm^3^ (200–900)	350
CD19 + cells/mm^3^ (100–500)	280
CD16 + 56 + cells/mm^3^ (60–600)	320

**Table 2:** Time sequence of clinical manifestations.

Year of catchment	Localization	Pathogen
1981	Inguinal and cervical lymph nodes	*M. kansasii*
1992	Lungs	Wrongly diagnosed as sarcoidosis
1993	Lymph nodes, Th-7, 8, 9, 11, 12, L1, 2, 5 left femur, maxilla, mandibula	*M. kansasii*
1995	Centre in the distal part of the left femur	*M. avium intracellulare* *M. gordonae*
1997	Granuloma in the right face	*M. lentiflavum*
1998	Left patella Distal part of the femur on the right Granuloma in the right face	*M. lentiflavum* *M. kansasii*
2001	Granuloma of the nasal septum	*M. avium intracellulare*
2002	Left knee	*M. flavescens*
2004	Sputum	*M. lentiflavum*
2007	Granuloma/nasal septum	*M. lentiflavum*
2009	Granuloma/nasal septum	*M. lentiflavum*
2013	Granuloma/nasal septum	*M. avium*
2014	TH 8, 11, L1, L5	*M. avium*
2016	Granuloma of the nasal septum	*M. lentiflavum*
2017	Colliquating granuloma in the nasal septum	*M. avium intracellulare*
2018	Granuloma of the nasal septum Colliquating granuloma in the right face	*M. avium* Start therapy rhIFN-*γ*
2020	Surgery of the nasal septum Granuloma of the right face healed	Microbiological investigation is negative
